# Chemical Composition, Bioactive Constituents, and Functional Value of Chinese Palm Fruit: Processing Effects, Nutritional Significance, and Industrial Prospects—A Review

**DOI:** 10.3390/foods15101618

**Published:** 2026-05-07

**Authors:** Eric Biney, Osei Belinda, Min Wang, Rui Li, Saiyi Zhong, Kit-Leong Cheong

**Affiliations:** 1 Guangdong Provincial Key Laboratory of Aquatic Product Processing and Safety, Guangdong Province Engineering Laboratory for Marine Biological Products, Guangdong Provincial Engineering Technology Research Center of Seafood, Guangdong Provincial Engineering Technology Research Center of Prefabricated Seafood Processing and Quality Control, College of Food Science and Technology, Guangdong Ocean University, Zhanjiang 524088, China; ericbi1831@gmail.com (E.B.); belindapokua16@gmail.com (O.B.); liruihn@163.com (R.L.); 2 College of Coastal Agriculture Sciences, Guangdong Ocean University, Zhanjiang 524088, China; wangmin@gdou.edu.cn

**Keywords:** Chinese oil palm, tocopherols, carotenoids, functional lipids

## Abstract

Palm oil and palm kernel oil are among the most widely consumed vegetable oils worldwide, but cultivar, agroecological conditions, and processing methods strongly influence their chemical properties. Although there is extensive research and production of palm oil in Southeast Asia, cultivation of its fruit in China, particularly in southern regions like Hainan and Yunnan, is severely underrepresented. This review critically summarizes current knowledge of the chemical composition, bioactive compounds, and functional properties of Chinese palm fruit components (both raw and processed), with a focus on processing-related changes and industrial applications. Current evidence suggests that Chinese palm mesocarp and kernel oils can be separated based on their general composition, fatty acid profiles, and minor lipids (such as tocopherols, carotenoids, and phytosterols), which are critical determinants of oxidative stability, nutritional quality, and processing functionality. Post-harvest practices (postmortem methods) and thermal processing strongly affect acid value, free fatty acid levels, and peroxide formation, with direct consequences for oil quality and refining efficiency. Chinese palm-derived lipids hold potential for functional foods, nutraceuticals, cosmetics, and bio-based materials used beyond their commonality as edible oil. Yet, gaps in cultivar-level chemical characterization, bioactive retention during processing, and evidence-based health evaluation remain. However, bridging these gaps using advanced analytical techniques and sustainable processing strategies will be of significant importance to endeavor towards the full utilization of Chinese palm fruit in both global food and bio-economy systems.

## 1. Introduction

Crude palm oil (CPO) and crude palm kernel oil (CPKO) from the fruit of oil palm trees produce two types of oils (palm and kernel), which are the most produced and used vegetable oils in the world, accounting for a large share of worldwide edible oil production [1,2]. The highest oil yield of oil palm, yielding several-fold more oil ha^−1^ than any other oilseed crop, coupled with its favorable physicochemical properties and low cost, has made palm oil the backbone commodity in the worldwide food system [3]. The unique fatty acid profile and room-temperature oxidative stability are valuable characteristics of palm oil; on the other hand, palm kernel is mainly used as a source of medium-chain saturated fatty acids and for its functional properties. Besides its food applications, these oils are very valuable raw material sources for foods, cosmetics, pharmaceuticals, the oleochemical industry (which supplies basic ingredients for consumer goods such as detergents and soaps), and countless other industrial uses [4]. Palm oil has traditionally been grown and processed—and studied scientifically in its environment—with an emphasis on the Southeast Asian nations that produce most of it, Indonesia and Malaysia. Consequently, most research on palm oil chemistry and nutritional composition, as well as palm oil processing technology, was conducted based on data collected from studies conducted with palms grown in equatorial tropical environments [5]. This regional concentration of research has led to a lack of knowledge about how the composition and functionality of palm oil might differ if sourced from non-traditional production regions. China has its own, increasingly significant role in global palm oil [6].

**Figure 1:**
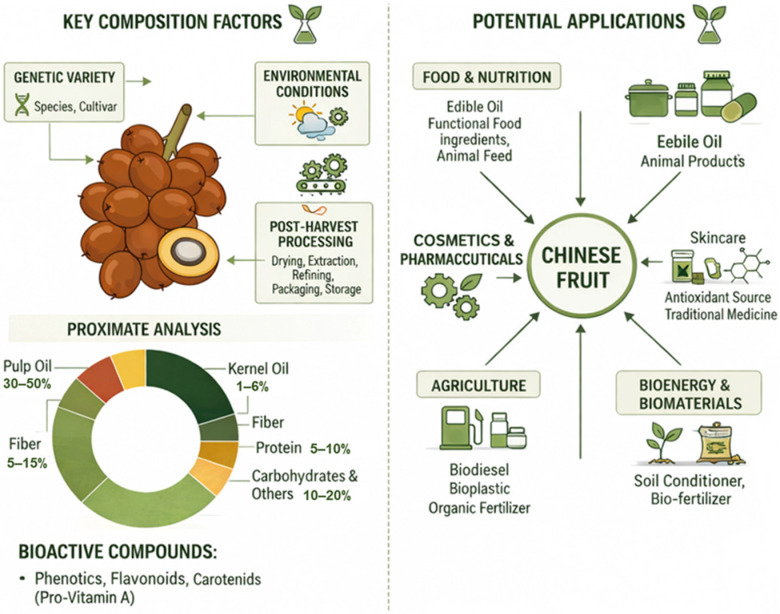
Chinese palm fruit: composition and application.

In addition, China’s dependent import has not been properly checked to ensure sufficient domestic consumption of palm oil, driven by rapid urbanization and population growth, as well as the expansion of food, cosmetics, and other industries (Figure 1) [3]. Mild tropical climate and favorable growing conditions in the south have enabled China to access small but crucial oil palm plantations concentrated predominantly in Hainan Province, where, on a local scale, there are limited patches of oil palm cultivation that are strategically significant. This allows for creating national oil security, reducing dependence on external imports, and establishing local palm oil value chains [7]. However, little is documented on the chemical composition, nutritional value, and functional properties of palm fruit grown in China compared to the numerous studies conducted on those of Southeast Asia. This is a significant gap, given that oil quality is not permanent, yet there was a large interaction effect between genetic and environmental factors and agronomic practices [8]. Selection of genotypes influences fruit structure, as reflected in both oil yield and lipid biosynthetic capacity, whereas environmental variables (i.e., temperature, rainfall distribution, and solar radiation) govern the fatty acid desaturation process and the accumulation of minor bioactive compounds [9]. The mineral composition of soil also affects enzyme and metabolic pathways involved in oil biosynthesis. It is important to note that factors affecting oil quality stability and bioactive retention include post-harvest handling and processing, such as harvesting regimens, sterilization techniques, and extraction methods [10,11]. Given the circumstances, palm fruit produced under Chinese agroecological conditions may differ from that produced in equatorial areas in terms of fatty acid profiles, mineral composition, and bioactive compounds such as tocopherols, carotenoids, and phytosterols [12]. These differences are significant and affect nutritional quality, oxidative stability, processing ease, and industrial utility. The merits and demerits of Chinese palm oil have so far not been discerned in an ordered characterization.

Therefore, it is high time to conduct a timely and critical review of the chemistry of this palm fruit. Such an assessment underpins a deeper understanding of regional diversity in palm oil composition for downstream (processing and refining) developments and local value addition. Moreover, further development of knowledge on the chemistry of Chinese palm fruit will help to better understand the diversity, quality, and functionality of palm oil globally, an emerging target for sustainable and resilient food and bio-based systems [13].

## 2. Botanical and Agronomic Background of Oil Palm

The African oil palm (*Elaeis guineensis* Jacq.), the most economically important palm species in the world, is cultivated for the commercial production of palm oil and palm kernel oil due to its high oil yield per hectare and broad tolerance to tropical/subtropical climates [14]. Oil palm is a perennial monocot in the family *Arecaceae*, and produces drupaceous fruits with three layers: an outer exocarp, fibrous oil-rich mesocarp, hard endocarp (shell), and kernel. Both mesocarp (oil palm) and kernel (palm kernel oil), which had different lipid profiles and functions in technology, were derived from oil [15].

The dominant oil palm variety in China, especially in Hainan Province, is Dura. Comparisons between Dura and Tenera highlight key differences in shell thickness, mesocarp proportion, and oil yield; in contrast, hemes are characterized by a moderate mesocarp-to-fruit ratio and oil yield relative to the extensively cultivated Tenera hybrids of Southeast Asia [16]. While the historical availability of planting material and early breeding activities probably explain the widespread use of Duras in China, its influence on oil yield efficiency and chemical composition is also likely to play a role [16,17]. In agronomic terms, Chinese oil palm (COP) has been grown in a totally different environment from that of equatorial Southeast Asia. Moreover, a higher biological or seasonal temperature decrease was occurring, including cool winter conditions that affect fruit development and lipid metabolism [18,19].

In contrast, during the critical drying period of ripening fruits, southern China exhibits a large seasonal rainfall distribution and relatively high interannual rainfall variability. The mineral composition of young natural oil-palm soils may also differ significantly (especially potassium, magnesium, and phosphorus) from that found in conventional palm cultivation areas. Relevant here are the actions of enzymes involved in oil biosynthesis [20].

It has been reported that environmental factors (temperature, light intensity, nutrient availability) may also regulate the major metabolic processes in oil palm fruit. Lipid (fatty acid) desaturation, which controls the proportion of saturated to unsaturated lipids, is sensitive to light and temperature [20,21]. Of note, the biosynthesis and accumulation of these minor but nutritionally significant materials (e.g., carotenoids, tocopherols) were conditioned on climate variables and soil fertility alike. The fatty acid profile and bioactive components of palm fruits cultivated in China may therefore differ from those found in a consistently hot and humid tropical area [22,23]. These gaps in botanical and agronomic traits suggest the need to conduct a regional characterization of Chinese palm fruit. Understanding the influence of local growing conditions on oil composition is important for optimizing processing practices and enhancing oil quality, thereby capitalizing on both domestic and global markets for palm-derived products [24].

## 3. Proximate and Mineral Composition of Palm Fruit Components

The palm fruit is a structurally and nutritionally complex matrix whose major components are the mesocarp and kernel, which in turn lead to varying proximate and mineral characteristics of technological and nutritional relevance [25]. Such differences are very crucial for better use of palm fruit beyond oil extraction. The mesocarp is the predominant lipid store of the palm fruit. It contains extremely high levels of crude fat, 45–55% (dry weight basis), moisture (fresh fruit): typically, 30–40% (fresh weight, FW) before processing, protein: ~2–5% (DW), carbohydrates (including dietary fiber): ~20–30% (DW), and ash: ~1–3% (DW) [26]. The high lipid content is the main reason for the economic importance of palm oil and its dominance in global vegetable oil markets, which consist of proteins, carbohydrates, dietary fiber, and minor amounts of lipids and residual moisture [27]. The mesocarp also contains other protein compounds that correlate with oil extraction efficiency, which is important for post-harvest stability. Mesocarp protein normally exists at low levels and is more representative of an energy reserve tissue than a nitrogen reserve [28].

For instance, palm kernel has a different proximate profile. The kernel is still considered an oil-rich commodity; however, it has a lower total lipid composition than the mesocarp, coupled with higher protein and ash proportions. While kernel proteins are relatively underutilized in human nutrition, they still confer functional properties to palm kernel cake used in animal feed formulations [29,30]. Also, a higher ash content indicates a greater presence of inorganic components; the matrix is expected to be more nutritionally enriched than the mesocarp [31]. Some fractions of palm fruit underwent mineral analysis and were found to contain K, Ca, Mg, P, Fe, and Zn as core macro- and micronutrients [32]. Potassium and magnesium also play essential roles in cell osmotic balance, enzyme activation, and lipid metabolism. But that does not mean calcium and phosphorus are any less important than the others; skeletal integrity of bones relies on them, as do cytosolic signaling mechanisms [33]. The trace elements, including Fe and Zn, are necessary for oxygen transport, the immune system, and antioxidation. A cross-examination shows that palm fruit components provide a moderate mineral balance when compared to the most common oilseeds, soybean, sunflower, and rapeseed. However, its mineral characteristics are generally ignored, as the major focus in palm oil research is on oil yield and fatty acid profile, among others. It is worth noting that if there are differences between mesocarp and kernel mineral content, soil fertility, fertilization methods, and agroecological conditions may influence these more than others [34,35].

Although not as significant as the lipid content, the mineral composition of palm fruit is important for its nutritional and functional uniqueness [36]. Besides serving as nutrients for humans, minerals are involved in the oxidative stability of products and enzyme-catalyzed reactions during palm processing. Moreover, by-products like palm kernel cake from mineral enrichment provide additional value to food and feed systems [37,38]. Therefore, a thorough understanding of the proximate and mineral composition would be required to enhance resource utilization efficiency [39,40].

## 4. Chemical Properties and Quality Indicators of Palm Oils

The chemical composition of palm oil and palm kernel oil has been identified as a key factor in evaluating their nutritional quality, technological properties, and industrial uses. The quality of palm oil is commonly evaluated using key chemical indices, including acid value (AV), free fatty acids (FFA), and peroxide value (PV). These parameters reflect distinct degradation pathways, namely hydrolytic and oxidative deterioration, and are strongly influenced by post-harvest handling and processing conditions [41]. Hydrolytic degradation results from enzymatic or moisture-induced hydrolysis of triacylglycerols (TAGs), releasing free fatty acids and partial glycerides. This process is primarily catalyzed by endogenous lipases present in the fruit mesocarp. Acid Value (AV) represents the amount of potassium hydroxide (mg KOH/g oil) required to neutralize free fatty acids. Free Fatty Acids (FFA) are typically expressed as % palmitic acid equivalents in palm oil. Freshly harvested and properly sterilized palm fruits typically yield crude palm oil with FFA levels of 3–5%, as shown in Table 1. In contrast, delayed processing can result in rapid increases in FFA levels due to lipase activity [42].

Such hydrolytic reactions may be negatively affected by post-harvest storage time, mechanical damage of the fruit skin, and even moisture or insufficient heat treatments (for sterilization). Studies on palm oil production in China show that improper post-harvest handling results in greater free fatty acid (FFA) accumulation, particularly when fruit processing is delayed and temperature control is poor. High-calorific residual oil is a consequence of an incomplete thermal inactivation of lipolytic enzymes during sterilization. It includes fatty acids resulting from the hydrolysis of triglycerides, which have low hedonic properties and are undesirable in the market [43,44]. Elevated Free Fatty Acid (FFA) levels are equally undesirable during refining because they increase alkali consumption and reduce oil output, thereby increasing production costs. Hence, better harvesting logistics and excellent heat treatment to control acid value (AV) and free fatty acid (FFA) are crucial for producing high-quality oil palm oils [45,46]. As one of the main parameters of primary lipid oxidation, it reflects the quantity of formed hydroperoxides in the primary stages of oxidative degradation. Palm oils are less oxidizable than other vegetable oils in general due to a higher amount of saturated fatty acids and also natural antioxidants tocopherols and carotenoids [45,47]. However, environmental factors such as oxygen, light, and high temperatures, as well as metal impurities during processing and storage, may accelerate peroxide formation. This value was also supported by Zhang et al., who assessed the effects of extraction technology, heat, and storage conditions on the peroxide value (PV) of Chinese palm oil [48]. Excess thermal stress and oil storage lead to higher peroxide values (PV), which affect shelf life and sensory acceptability. While regularly monitoring the peroxide value helps control oil degradation and overall food quality, it also helps ensure oil stability and safety, as well as the stability and safety of a wide variety of foods like fried or baked goods [49].

The international quality parameters for palm oil comprise maximum limits of acid value (AV), free fatty acid (FFA), and peroxide value (PV), stipulated and harmonized by institutions such as Codex Alimentarius [50]. Comparative studies show that when the best post-harvest and processing practices are employed, palm oils processed from Chinese-raised fruits can comply with, or be nearly compliant with, most global standards. This shows the technical feasibility for producing globally competitive qualities of palm oils in China. However, chemical quality indices still provide valuable information for assessing the influence of agronomic practices, post-harvest handling, and processing on palm oil quality. Systematic regulation of AV, FFA, (See Table 2), and PV is crucial to achieve greater cost-effectiveness and stability in product quality, as well as in the nutritional and commercial quality of palm oils produced by Chinese plantations [51,52].

**Table 1:** Composition and quality indicators of Chinese palm fruit systems.

Region (China)	Cultivar	Matrix	Processing/Extraction Method	AV (mg KOH/g)	FFA (%)	PV (meq O_2_/kg)	Major Fatty Acids (%)	Bioactive Compounds (mg/kg)	References
Hainan	Dura	Mesocarp oil (CPO)	Steam sterilization + mechanical pressing	6–10	3–5	<10	C16:0 (40–45), C18:1 (35–42), C18:2 (8–10)	Tocopherols/Tocotrienols: 600–900; Carotenoids: 500–700	[53]
Hainan	Tenera	Mesocarp oil (CPO)	Steam sterilization + screw press	5–8	2–4	<8	C16:0 (39–44), C18:1 (36–43), C18:2 (9–11)	Tocotrienols dominant; Carotenoids: 450–650	[54]
Yunnan	Dura	Mesocarp oil	Small-scale processing (delayed handling)	8–15	4–8	8–15	C16:0 (40–46), C18:1 (34–40), C18:2 (7–10)	Reduced carotenoids due to handling losses	[55]
Hainan	Mixed	Kernel oil (PKO)	Mechanical pressing	2–5	1–3	<5	C12:0 (45–52), C14:0 (14–18), C16:0 (8–10)	Tocopherols: 50–100; negligible carotenoids	[56]

**Table 2:** Processing effects on quality indicators and bioactive retention in palm oil.

Processing Stage	Condition	Effect on AV/FFA	Effect on PV	Effect on Fatty Acids	Effect on Bioactive Compounds	Key Mechanism	References
Postharvest delay	>24–48 h	↑ Significant increase	Minor	No major change	Slight decrease	Lipase-mediated hydrolysis	[57]
Sterilization	120–140 °C steam	↓ FFA formation	Minimal	No change	Moderate carotenoid loss (if excessive)	Enzyme inactivation	[58]
Mechanical extraction	Pressing	Neutral	Slight ↑	No change	No change	Oxygen exposure	[59]
Bleaching	Adsorbent clay	No effect	↓ PV	No change	↓ Tocopherols, ↓ Carotenoids	Adsorption	[60]
Deodorization	200–260 °C	Slight ↓	↓ PV	Minor isomerization	Significant loss of vitamin E & carotenoids	Thermal degradation	[61]
Storage	Heat + oxygen	Slight ↑	↑ PV	No change	Gradual degradation	Oxidation reactions	[62]

The arrow showing up means an upwards regulation whiles the arrow showing down means downwards regulation.

## 5. Fatty Acid Composition and Lipid Structure

**Figure 2:**
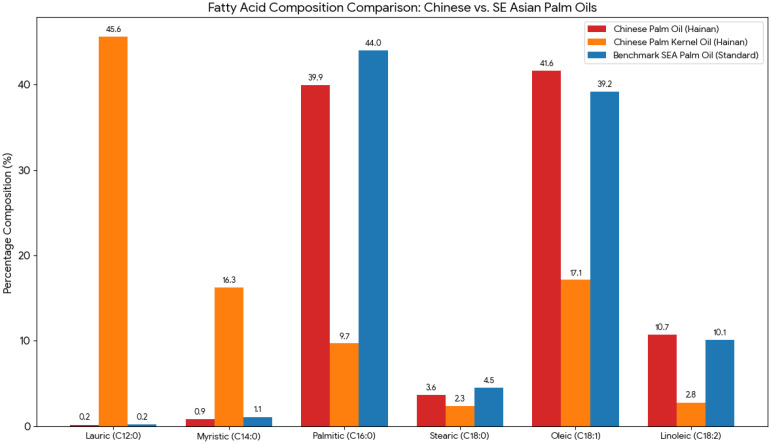
The comparison of the fatty acid composition of palm oil and palm kernel oil produced in China (specifically from Hainan plantations) against a benchmark Southeast Asian palm oil (representing standard production from Malaysia or Indonesia) [69].

Lipid composition is the primary determinant of the nutritional value, oxidative stability, and functional behavior of palm fruit oils. The fatty acid composition and lipid structures in the palm oil (PO)–palm kernel oil (PKO) system differ significantly, as they originate from the mesocarp and kernel, respectively [56,63]. Palm kernel oil, on the other hand, is from fat and leaves behind mainly lauric acid (C12:0), but also some myristic (C14:0) and palmitic (C16:0) acids (shown in Figure 2). Palmitic acid (C16:0) was the major saturated fatty acid and contributed 40–45% of the total fatty acids, followed by stearic acid (C18:0). The main unsaturated fatty acid is oleic acid (C18:1), at around 40–45%, and small amounts of linoleic acid (C18:2) [63,64]. The balance between saturated and monounsaturated fatty acids gives both stability and health potential, due to the beneficial properties of oleic acid with respect to lipid metabolism and cardiovascular risk [65]. The mesocarp fraction of palm oil has nearly equal amounts of saturated and unsaturated fatty acids [66]. These types of fat include medium-chain triglycerides (MCTs), which are metabolized differently from long-chain fatty acids, with rapid oxidation and various physiological and nutritional functions, including possible antibacterial effects; MCTs are also suitable for some foods and personal care items [67,68].

These types of fat include medium-chain triglycerides (MCTs), which are metabolized differently from long-chain fatty acids, with rapid oxidation and various physiological and nutritional functions, including possible antibacterial effects; MCTs are also suitable for some foods and personal care items [70]. For example, palm oil’s high saturated fat content means it produces oil with good oxidative stability and heat tolerance, making palm oil products widely used in frying, cooking, food frying, and general oil processing applications where fats need to be stable [71]. Palm kernel oil is unlike all edible fats and oils in that its medium-chain-length saturated triglyceride has a low melting point and rapid digestibility; the tart heat profile gives it unique texture and functional properties for both food and non-food applications. The lipid form also prevents the emulsion and solid fat breakdown, allowing it to be used in bakery fats, confectionery, and specialty fats [72].

Chinese palm oil vs. SEA benchmarks: Chinese palm oil (Hainan) generally has a lower saturated fat profile than the Southeast Asian benchmark. It contains less Palmitic acid (C16:0) (approx. 39.9 vs. 44.0%) and a higher level of Oleic acid (C18:1) (ca. 41.6 versus 39.2%) [73]. Palm kernel oil (PKO) vs. palm oil (PO): PKO is another type of oil with exactly the opposite chemical composition compared to PO, but it comes from the same fruit. It is a lauric oil; lauric acid (C12:0) comprises approximately 46% of its content, as illustrated in Figure 2 [74]. Saturated vs. unsaturated—regular palm oils (Chinese/SEA), especially coconut oil, have almost equal proportions of saturated (palmitic, stearic) and unsaturated (oleic, linoleic) fats.

On the other hand, palm kernel oil is far more saturated (over 80% of these fatty acids are saturated). Palm oil and palm kernel oil, which are both high in saturated fatty acids and low in unsaturated fatty acids, should be considered in the complete food plan alongside physiological needs. While traditionally strong links have been found between saturated fats and cardiovascular risk, palm oil nonetheless provides a delicate balance of stability vs. health without any health concerns, as its ecosystem contains monounsaturated oleic acid and minor polyunsaturated fatty acids [75]. The medium-chain triglyceride (MCT) profile of palm kernel oil can serve as another potential energy source and provide scope for clinical nutrition, functional foods, and nutraceutical formulations [76].

## 6. Minor Bioactive Constituents

### 6.1. Tocopherols and Vitamin E

Palm oil is rich in vitamin E (tocotrienols and tocopherols), which has a considerably high antioxidant importance. These lipophilic compounds are primarily located within the oil fraction and can provide oxidative stability, inhibit the peroxidation of polyunsaturated fatty acids, and protect the cell membranes of biological systems [77,78]. Tocotrienols, in particular, which are more abundant in palm oil than any other vegetable oils, showed even higher antioxidant activity due to the simple presence of unsaturated side chains and thus greater radical-scavenging efficiency. In palm oil, tocopherol/tocotrienol levels and stability are strongly influenced by the processing system used, along with the degree of refining [79]. The bioactive lipids can also be co-extracted during the crude extraction of the oil process by mechanical pressing or solvent extraction at sufficient degrees to develop bioactivity; however, further refining processing, such as degumming, neutralization, bleaching, and deodorizing, occurs, which often results in tocopherol loss due to the high temperature involved and contact with chemicals while retaining contact periods with oxygen. For this reason, it is crucial to tightly control extraction and refining conditions to obtain a high vitamin E content with acceptable oil quality [80]. Besides their protective action against oxidative stress, tocopherols and tocotrienols have been associated with a range of health-promoting effects, including anti-inflammatory, neuroprotective, and cardioprotective effects. The presence of regional oils, such as palm oil in China, suggests these edibles may serve as more relevant functional food components, acting as nutraceutical agents or dietary supplements. Processing methods need to be optimized to improve bioactive compound retention and increase the nutritional value and shelf life of a palm-based product and its activity [81].

### 6.2. Carotenoids (β-Carotene)

Crude palm oil is rich in carotenoids, such as β-carotene, which give the oil its red-orange color. β-Carotene functions as a provitamin [82]. Palm oil contains carotenoids, notably β-carotene, which function as provitamin A compounds by serving as metabolic precursors of retinol in animals. This property makes the oil particularly important for public health, especially in mining areas. Vitamin A malnutrition persists because the intake of β-carotene-rich palm oil can overcome micronutrient deficiencies and associated health issues, such as night blindness and immunodeficiency [83]. Factors such as fruit maturity, variety, environmental conditions, and post-harvest practices will also affect carotenoid contents and stability in palm oil. The highest levels of β-carotene are found in fully mature mesocarp tissues, and poor harvesting practices or delays in processing can initiate breakdown, leading to subsequent loss from the diet [84,85]. Moreover, carotenoids are sensitive to heat, light, oxygen, and acid, so their conservation throughout the extraction, clarification, and refining processes is a huge techno-economic challenge. Practical strategies for industrial storage and processing, such as conserving carotenoids, gentle heat treatments, low bleaching level, a refined storage regime based on the carotenoid profile, and limited exposure to oxygen/no light [86]. Having some quantity of β-carotene remains will increase the internal value (nutritional) and also provide color as well as antioxidant properties to palm oil [87]. That means improving processing with a lean toward β-carotene retention in Chinese palm fruit represents a vital step in the development of nutritionally enriched, functionally active palm oil, potentially because regionally identified cultivars and agronomic conditions affect carotenogenic pathways [88,89].

### 6.3. Phytosterols

Phytosterols are a subclass of sterols, which are plant-derived compounds with structures similar to cholesterol and natural constituents of the mesocarp and kernel fractions in palm oil. Palm oil was shown to have the highest concentration of β-sitosterol, campesterol, and stigmasterol [90]. Their diversity of effects can improve the functional quality of palm oil (PO) for health by modifying cholesterol metabolism. The main physiological activity of phytosterols is the lowering of blood cholesterol through competition-based inhibition of intestinal cholesterol absorption. Unesterified plant sterols are described to reduce circulating low-density lipoprotein cholesterol (LDL-C) concentrations to support cardiovascular health, for instance, through the partial displacement of dietary and biliary cholesterol from micelles within the small intestine [90,91]. Thus, the palm oil’s bioactive role indicates that it has not only a food fat but also a potential functional food ingredient with nutraceutical significance. Phytosterol quantity and bioactivity vary based on the types of oil extraction, pre-treatments, and refining processes [92]. While phytosterol content can be preserved through mechanical and cold-press processes, excessive refining, bleaching, and deodorization can lead to partial degradation or loss. This necessitates the development of processing methods that retain maximal levels of these bioactive sterols [93]. Data on critical aspects, such as phytosterol content and stability, for Chinese palm oil are comparatively under-documented compared to Southeast Asian varieties. This maintenance of phytosterols improves the nutritional and practical properties of palm oil, increasing its potential as an ingredient for health-promoting food products, enriched oil brands, and other value-added applications within the functional food and nutraceutical industry [92,93].

## 7. Processing Effects and Technological Considerations

**Figure 3:**
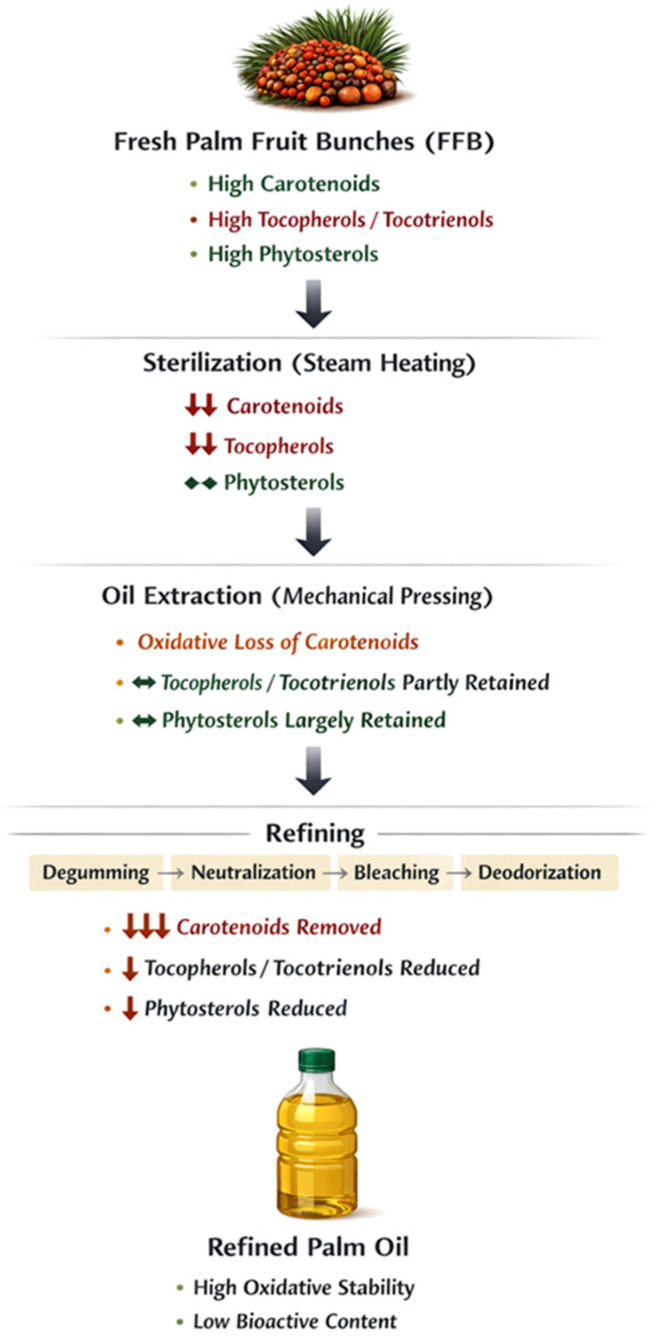
Impact of palm oil processing on bioactive compounds: from fresh fruit bunches to refined oil.

Post-harvest processing of palm oil (PO) and its processing technologies have a decisive impact on PO’s quality, nutritional value, and functional properties. Thermal sterilization, mechanical extraction, and storage are key steps that significantly affect oil composition, stability, and bioactive content [94]. Sterilization is the first critical unit operation in palm oil processing, wherein freshly harvested fruit bunches undergo steam treatment before mechanical digestion. The temperature–time profile of this thermal treatment must be carefully optimized, as excessive heat or prolonged exposure can degrade thermolabile bioactive constituents, such as carotenoids and tocopherols, thereby reducing antioxidant activity and nutritional quality (as shown in Table 2) [95]. In contrast, insufficient sterilization causes home lipase to be excessively inert, and free fatty acid (FFA) is formed quickly, leading to increased acid value (AV), which will affect the quality and refining efficiency of oil. Thus, the efficiencies of mechanical pressing or expeller extraction, and the bioactive residual in oil, are regulated [96]. This optional pressing condition uses a simple oxidative stress balance, combined with complex oxidative stress and thermal decomposition of the material, to maximize oil extraction. It indicates that minor bioactive components, color, and oxidative stability may be compromised due to over-pressuring or frictional heat generated during extraction (Figure 3) [97]. However, the post-extraction method used when oil is stored also plays an important role, as it can lead to deterioration in oil quality. The oxidation of fats can be accelerated when lipids are exposed to light, oxygen, and metallic ions at elevated temperatures, resulting in a high peroxide value (PV) that significantly reduces functional shelf-life [98]. Other factors included the role of moisture content and the integrity of the package container on oxidative stability and microbial safety [99].

Novel technological advances can enhance oil quality and nutritional or functional attributes. These factors are: (I) gentle heat treatments capable of accomplishing the balance between enzyme inactivation and preservation of bioactivity [100,101]; (II) ready-to-eat logistics (temperature, inert gases, opaque containers); (III) enzyme inhibitors or fast post-harvest FFA (free fatty acid) production without high heat treatment [102]; (IV) optimized extraction techniques, such as cold pressing and low-temperature expelling, which can yield higher tocopherol, carotenoid, and phytosterol contents [103].

## 8. Nutritional, Functional, and Industrial Applications

**Figure 4:**
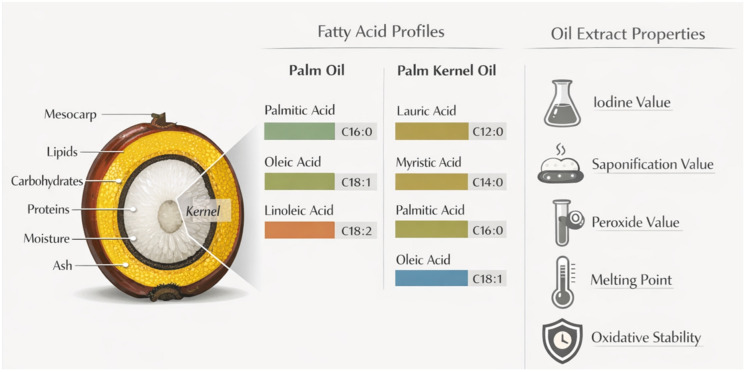
Chemical composition of Chinese palm fruits (*Elaeis guineensis*) and oil extraction properties [98].

According to [104,105,106]. These edible oils have a composition and use similar to palm fruit (Chinese) oil and palm kernel oil. Due to their unique fatty acid profiles, advanced lipid structures, and bioactive compounds, they are considered promising ingredients for food, health (nutraceutical), and industrial applications (Figure 4).

The balanced ratio of saturated to unsaturated fatty acids in palm oil, along with its distribution among tocopherols, tocotrienols, carotenoids, and phytosterols, makes it a significant source of energy and bioactivity [107]. These features provide antioxidant properties for cardiovascular protection and vitamin A, making palm oil more crucial in fortified foods and dietetic formulations. Because PKO is a source of medium-chain fatty acids, it provides an immediate source of energy. It has potential clinical use, for example, in patients with fat malabsorption who require an easily absorbable or metabolizable lipid [108]. The presence of synergistic bioactive components in Chinese palm oils, such as vitamin E, β-carotene, and phytosterols, provides functional benefits. This highlights the supplemental antioxidant activity, cholesterol-lowering activity, and immune modulation activity of the compounds and justifies their use in nutraceutical products, fortified oils, or functional foods [109]. Moreover, the semisolid nature of palm oil at ambient temperatures enables its direct use in spreads, margarines, and bakery fats without hydrogenation, to maintain palatability. The use of palm oil extends beyond food applications, but takes advantage of its physicochemical properties [110]. The high oxidative stability and heat stability of palm oil make it suitable for cosmetic applications, pharmaceutical creams, and topical products.

In contrast, the emulsion-stability properties of palm kernel oil are relevant in detergents, soaps, and personal care products. This is particularly noteworthy, as they are both precursors to bio-based materials, such as biodegradable lubricants, oleochemicals, and biofuels, showcasing their multiplicative industrial potential [111]. Alternatively, Chinese palm oils are a valuable raw material that can provide not only nutritional but also functional applications. Thus, the combination of agronomic, chemical, and technical knowledge is important for these applications [112].

Development of the oil palm industry in China may provide a strategic opportunity to curtail over-reliance on imported palm oil, improve domestic production capacity, and promote the development of the regional bioeconomy in southern provinces (i.e., Hainan and Yunnan) [113]. Local production is not just an issue of national food security; it also presents opportunities for value-added industries, including functional foods, nutraceuticals, and bio-based materials. Despite these market opportunities, Chinese oil palm operations face numerous sustainability challenges [114], including constraints on land use, water use, and soil fertility, as well as limited availability of high-yielding and disease-resistant cultivars and inefficient processing technologies (which may affect bioactives retention), etc. If state incentives are poorly managed, this crop expansion can harm local ecosystems and resource use [115].

## 9. Research Gaps

The knowledge gaps that have emerged pose an urgent need to be addressed for a sustainable and competitive palm oil sector in China. These are cultivar development and breeding to produce cultivars adaptable to district conditions with higher oil yield, a balanced fatty acid profile, and bioactive content. The processing optimization: These are post-harvest management and extraction techniques that can help optimize oil quality with minimal energy/chemical consumption [116]. Bioactive retention strategies: Technological solutions for tocopherols, carotenoids, and phytosterols retention during processing and storage. Life cycle assessments: Environmental impact, water footprint, and greenhouse gas emissions stemming from growing and processing [117].

Furthermore, to meet the precepts of globalized sustainable food systems, the Chinese palm industry should use land sustainably through practices such as efficient energy use and environmentally friendly practices. This can increase resource productivity, reduce environmental pressure, and maximize the social returns from smallholder farmers and local communities. With targeted research and technological breakthroughs, Chinese palm fruit has the potential to serve as a bioactive-rich, high-quality oil resource that can meet national demand, be widely utilized for functional/industrial applications, and support a sustainable production scheme [118]. Investments in breeding programs, ‘green’ extraction technologies, and quality assurance systems will be critical for the industry to sustain itself and earn its rightful place as a responsible, nutritionally valuable source of oil on the world stage [119].

## 10. Conclusions

Chinese palm fruit is a relatively under-studied and still an untapped resource; it has tremendous potential in both domestic and international markets. One of the defining features of argan oil’s nutritional and functional properties is its specific chemical composition, which includes a relatively high content of saturated and unsaturated fatty acids, as well as bioactive compounds such as tocopherols, carotenoids, and phytosterols. These characteristics make Chinese palm oils a promising ingredient not only for food applications but also for functional foods, nutraceuticals, cosmetics, and bio-based materials. For a comprehensive understanding of the effects of local cultivars, agronomic conditions, and post-harvest practices on overall oil quality, such characterization should be performed in individual regions to assess proximate composition, mineral content, fatty acid profiles, and minor bioactives. Likewise, the mind develops operating procedures capable of reducing oxidative damage and providing the oils a quality higher than international standards, respectively, sustainable for industrial use. Overall, Chinese palm fruit is a multifunctional resource with the potential to promote food security, industrial development, and sustainable agriculture. The key sector for gaining strength, becoming independent, and developing its potential will be the strategic alignment of agronomic, chemical, and technological properties that meet international standards, so as to contribute positively to a sustainable palm oil economy in a competitive manner within both national and international palm oil markets.

## Data Availability

No new data were created or analyzed in this study. Data sharing is not applicable to this article.

## Abbreviations

**Abbreviations**	**Meaning**
PO	palm oil
PKO	palm kernel oil
COP	Chinese oil palm
AV	acid value
FFA	free fatty acid
PV	peroxide value
MCT	medium-chain triglycerides
LDL-C	low-density lipoprotein cholesterol
FFB	Fresh Palm Fruit Bunches
